# Eye movements while judging faces for trustworthiness and dominance

**DOI:** 10.7717/peerj.5702

**Published:** 2018-10-11

**Authors:** Frouke Hermens, Marius Golubickis, C. Neil Macrae

**Affiliations:** 1 School of Psychology, University of Lincoln, Lincoln, Lincolnshire, UK; 2 School of Psychology, University of Aberdeen, Aberdeen, UK

**Keywords:** Eye movements, Social judgments, Face perception

## Abstract

Past studies examining how people judge faces for trustworthiness and dominance have suggested that they use particular facial features (e.g. mouth features for trustworthiness, eyebrow and cheek features for dominance ratings) to complete the task. Here, we examine whether eye movements during the task reflect the importance of these features. We here compared eye movements for trustworthiness and dominance ratings of face images under three stimulus configurations: Small images (mimicking large viewing distances), large images (mimicking face to face viewing), and a moving window condition (removing extrafoveal information). Whereas first area fixated, dwell times, and number of fixations depended on the size of the stimuli and the availability of extrafoveal vision, and varied substantially across participants, no clear task differences were found. These results indicate that gaze patterns for face stimuli are highly individual, do not vary between trustworthiness and dominance ratings, but are influenced by the size of the stimuli and the availability of extrafoveal vision.

## Introduction

When presented with a person’s face, people rapidly form first impressions (Ballew & Todorov, 2007; Bar, Neta & Linz, 2006; Todorov, Pakrashi & Oosterhof, 2009; Willis & Todorov, 2006). These first impressions have been shown to have real life implications, for example, in elections (Ballew & Todorov, 2007; Hall et al., 2009; Todorov et al., 2005), although there is no clear support for their validity (Efferson & Vogt, 2013; Zebrowitz et al., 1998). While many different social judgments can be made about other people (e.g. competence, friendliness, approachability), research has suggested that these can be reduced to two main, largely independent dimensions: trustworthiness and dominance. Together, these dimensions explain around 80% of the variance in people’s ratings of another person’s face on various social dimensions (Oosterhof & Todorov, 2008; Todorov et al., 2008). When computer-generated face stimuli are created based on these ratings, the generated faces suggest that smiles lead to trustworthy faces, whereas expressions of anger lead to untrustworthy faces. Dominance was signalled by strength and masculinity (Oosterhof & Todorov, 2008).

A more detailed understanding of the facial features associated with trustworthiness and dominance was obtained by analysing ratings for a large set of ambient facial images (Vernon et al., 2014), which can be expected to vary more strongly on the above features than a set of standardized faces as typically found in a face database (Ma, Correll & Wittenbrink, 2015; Langner et al., 2010). This analysis confirmed trustworthiness and dominance to be the two main dimensions (Vernon et al., 2014), and identified a third dimension (‘youthfull-attractiveness’, Sutherland et al. (2013)). Facial features important for these dimensions were identified by coding landmarks for each face and extracting facial features (such as head width and eyebrow width) from these landmarks (Vernon et al., 2014). Trustworthiness ratings were most strongly linked to the mouth shape, and included features such as mouth area, mouth height, and bottom lip curve, in agreement with earlier observations that trustworthiness is related to a smiling expression (Oosterhof & Todorov, 2008). Features most strongly related to dominance ratings were those showing a masculine appearance, and included eyebrow height, cheek gradient, and skin saturation, also in agreement with Oosterhof & Todorov (2008). The third, youthful-attractiveness dimension most strongly related to eye features, such as eye area, iris area, and eye width (Vernon et al., 2014). Overall, however, there was no single dominant facial feature that predicted trustworthiness or dominance, and ratings were best predicted by a combination of features.

When presented with a face stimulus, people tend to make eye movements for detailed processing of the eyes, mouth region, and nose (Barton et al., 2006; Walker-Smith, Gale & Findlay, 1977). From studies of eye movement in reading, it is known that for detailed processing of stimulus features, items need to be foveated. Reading without a fovea is difficult (Rayner & Bertera, 1979), and reading at normal speed requires a window of at least 12–15 characters right of fixation (McConkie & Rayner, 1975). Another processing limitation in extrafoveal vision is crowding (Whitney & Levi, 2011), where nearby features interfere with the accurate identification of the features of a target object. Crowding occurs for facial features (Martelli, Majaj & Pelli, 2005), and it suggests that in order to process faces for trustworthiness and dominance, the relevant features need to be foveated. The question therefore arises whether eye movements during trustworthiness and dominance ratings reflect those features of the face associated with the two dimensions.

Past studies examining such task-related eye movement strategies when judging faces have yielded inconsistent results. Yarbus (1967) found that his own eye movements towards a painting strongly depended on what question he asked himself about the painting, an observation that was confirmed in larger sets of participants (DeAngelus & Pelz, 2009; Tatler et al., 2010), and with the exception of one study (Greene, Liu & Wolfe, 2012), the task of the observer could be predicted from their eye movements (Borji & Itti, 2014; Haji-Abolhassani & Clark, 2014; Kanan et al., 2014, 2015). When judging faces for emotions, the pattern of eye movements reflects the judged emotion (Schurgin et al., 2014), and eye movements depended on whether two faces where matched for identify or for expression (Malcolm et al., 2008), or whether the more feminine face, the same identity or the same gender was decided upon (Armann & Bülthoff, 2009). Likewise, eye movements differed when judging faces for identity, gender, or performing an emotional state task (Peterson & Eckstein, 2012). A recent study also found a higher fixation density on the mouth for happiness judgments of happy faces, compared to trustworthiness ratings (Calvo, Krumhuber & Fernández-Martn, 2018). In contrast, Pelphrey et al. (2002) found no differences in eye movement patterns between free viewing and judging emotion, and no differences in eye movements were found between judging faces for age and attractiveness (Kwart, Foulsham & Kingstone, 2012), or fatigue and attractiveness (Nguyen, Isaacowitz & Rubin, 2009). None of these studies, however, have focused on trustworthiness and dominance ratings. Because the two ratings have been shown to be largely independent (Oosterhof & Todorov, 2008; Todorov et al., 2008), one could possibly expect stronger task differences than for tasks that may be psychologically related.

The inconsistency of task influences on eye movements during face processing may suggest that eye movements are not critical for the task, and that faces can be processed holistically by fixating on the eyes and the mouth and nose areas. However, when eye movements were restricted during face processing, learning was impaired (Henderson, Williams & Falk, 2005). Moreover, instructing a patient with a deficits in emotion recognition to adopt a more typical gaze pattern towards faces removed the deficit (Adolphs et al., 2005). For autism it has been suggested that differences in gaze patterns towards faces are an important reason for the observed emotion recognition problems (Pelphrey et al., 2002). In contrast, however, face recognition seem to require only two fixations on the face, when aimed at the nose region (Hsiao & Cottrell, 2008), and attractiveness ratings for faces were the same for foveal and peripheral presentation of the faces (Guo, Liu & Roebuck, 2011). Together, the literature on task effects during face perception does not seem to provide a clear answer to our question whether eye movements in trustworthiness and dominance ratings reflect the features used for the different tasks.

In the present study, we systematically compare eye movements while people judged faces for trustworthiness and dominance. To avoid biasing eye movements by facial expressions (Pollux, Hall & Guo, 2014; Schurgin et al., 2014), we used standardized images from the Radboud Face Database (Langner et al., 2010), each having a neutral expression. We varied the way the faces were presented to mimic possible differences in viewing distances (large viewing distances mimicked by small face images, and face-to-face viewing mimicked by large face images). We also included a condition in which extrafoveal information was blocked using a moving window paradigm (only the area around the current fixation visible) to examine whether more extensive scanning of the faces can be found if extrafoveal information is no longer available. These different presentation modes were explored in separate experiments, but we will present them here side-by-side, allowing for comparisons of eye movement patterns for different image sizes and presentation modes. If detailed processing of facial features is important for trustworthiness and dominance ratings, we expect participants to differentially fixate these regions in the two tasks (Oosterhof & Todorov, 2008; Vernon et al., 2014). As a consequence, we expect more and longer fixations on the mouth for trustworthiness ratings, and more and longer fixations on regions signalling dominance for dominance ratings (e.g. cheek, chin, and eye-brow features). In contrast, if the two tasks rely on holistic processing of the face, no systematic effects of task on eye movements may be found, in line with past studies showing only small task effects in face viewing (Armann & Bülthoff, 2009; Kwart, Foulsham & Kingstone, 2012; Malcolm et al., 2008; Nguyen, Isaacowitz & Rubin, 2009; Pelphrey et al., 2002; Peterson & Eckstein, 2012; Schurgin et al., 2014). How image size influences task effects on eye movement patterns is not immediately clear from the literature. Image size itself, is expected to influence eye movement patterns, with a stronger focus on the centre of the face for smaller images (Guo, 2013). If face processing is holistic, one may expect eye movement patterns to depend on the availability of extrafoveal vision. However, based on a direct comparison between foveal and peripheral beauty judgments, which saw no effect of presentation mode (Guo, Liu & Roebuck, 2011), there may be no effect of restriction of extrafoveal vision on ratings or eye movement patterns.

## Methods

### Participants

Three groups of participants were tested, each performing trustworthiness and dominance ratings for the same set of faces, but under three different viewing conditions. The first group of participants, who rated small images of the faces (mimicking viewing at a distance), consisted of 20 participants (six male, aged between 21 and 38 years), were recruited by word-of-mouth and took part without reimbursement. A total of 21 further participants (three male, sampled from first and second year psychology students) rated the large images of faces (mimicking face-to-face viewing). These participants were recruited via an online participant recruitment system and took part in return for course credit. Another 21 participants (three males, sampled from first and second year psychology students) rated the images with a moving window (removing extrafoveal information). These participants were also recruited using the online participant recruitment system, and also received course credit for taking part. Recruitment and reimbursement differed between the first and the latter two groups, but based on past experience with different recruitment methods and reimbursements within a single set of experiments (and with consistent results, Hermens, Bindemann & Burton (2017)), we do not expect these differences to have an effect. Participants all provided written consent for the study that was approved by the local ethics committee (University of Aberdeen, UK), in agreement with guidelines of the British Psychological Society and the declaration of Helsinki.

We based the number of participants per viewing condition on typical numbers of participants in eye tracking studies in general and past studies of task effects on eye movements (around 20 per condition). A recent study has suggested that for the mixed effects models that we are applying, there should be around 1,600 observations per condition (Brysbaert & Stevens, 2018). Per viewing condition, we had around 800 observations per condition (20–22 participants times 39 images), but across the three viewing conditions (assuming that stimulus size and viewing condition does not interact with task), sufficient observations per task were obtained (2,457).

### Apparatus

A Dell 19 inch flat screen (1280 by 1024 or 1024 by 768 pixels resolution, at a 60 Hz refresh rate) was used to present the stimuli to the participants. The screen was positioned at a distance of 77 cm from the observer (controlled using a UHCOT Tech Headpot standard chin rest). A Dell Optiplex PC with a dual core processor, running the Windows XP operating system and software created with SR Research’s Experiment Builder, was used to control stimulus presentation. Eye movements were recorded using an Eyelink 1000 desk-mount system at a sampling rate of 1,000 Hz, using the combined pupil centroid and corneal reflection setting. A standard USB mouse was used to collect participants’ responses.

### Stimuli

**Figure 1:**
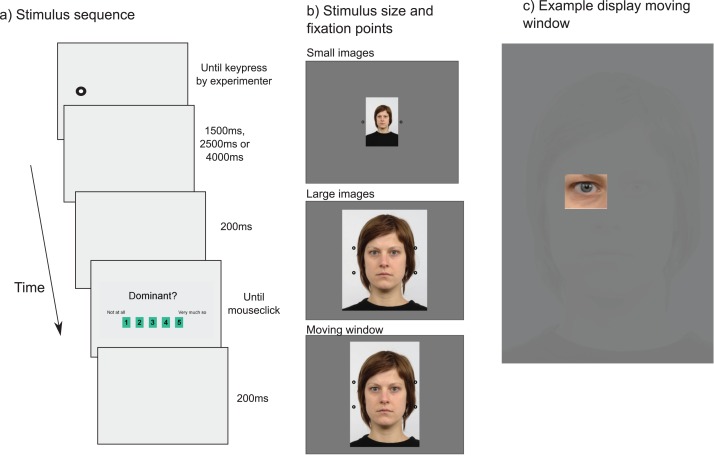
(A) Trial sequence. Participants first fixated a drift correction target, presented either to the left or the right of the face, which was replaced (after confirmation of fixation by the experimenter) by a centrally presented face image. The image was presented for 1,500 ms (smaller images), 2,500 ms (larger images), or 4,000 ms (moving window), followed by a short blank screen (200 ms) and a rating scale. Participants used the computer mouse to indicate their response by clicking one of the response boxes shown. (B) Illustration of the size and position of the images within the screen for each of the three image size conditions, together with the locations of the initial fixation points, aimed to avoid biasing participants’ eye movements to certain regions of the image (note that in this illustration the background is shown as black, while in the actual experiment it was grey). (C) Example of the moving window condition, where the highly visible part of the image moved with the direction of gaze of the participant (in the example, the participant was looking at the left eye). In the background, a low contrast version of the original image was presented as a reference as to where to find the face stimulus.

Examples of stimuli are shown in Fig. 1. A total of 39 face images (20 males) were selected from the Radboud Faces Database (Langner et al., 2010), each showing a Caucasian face with a neutral expression. These images were presented to three groups of participants under different viewing conditions, as illustrated in Fig. 1B: A small image condition, a large image condition, and a moving window condition (see Fig. 1C). In each of these conditions, images were presented in the centre of the screen, with initial fixation points presented laterally (left and right) to force participants to saccade into the face image.

In the small image condition (mimicking large viewing distances), the images were all scaled to 272 by 410 pixels (presented inside a display of 1280 by 1024 pixels) corresponding to a size of 6.0° by 8.9° of visual angle. In the large image condition (mimicking face-to-face viewing), images were cropped to enhance the size of the face region, showing just the area from the top of the face to the bottom of the neck. In the condition, stimuli were of around 530 by 683 pixels in size (and presented in a display of 1024 by 768 pixels), corresponding to around 13° by 23° of visual angle. For the moving window presentation similarly cropped images were used, but now the size was kept constant at 480 by 650 pixels (12.5° by 25.0° of visual angle within a display of 1024 by 768 pixels). The moving window (Fig. 1C) consisted of an area of 4.2° by 5.5° of visual angle and showed a high resolution section of the image where the participant looked (gaze contingent window), continuously updated from the eye movement recordings (controlled by SR Research’s Experiment Builder software). To give the participants some indication of where to find the face on the screen, the area around the window was set to a extremely faint version of the original image (Fig. 1C), for which the brightness of the original image was decreased by 150 units, the contrast by 125 units, and the saturation by 245 units using the Irfanview image editing software.

While past work has suggested that 100 ms suffice to form first impressions that are consistent across observers (Willis & Todorov, 2006), we did not expect to find meaningful patterns of eye movements during such a short interval, as reading research has suggested that fixations during reading and image exploration take around 200–250 ms each (Rayner, 1998). While it would be possible to present the stimuli until participants provided a response (e.g. by a scale presented below the image), it was decided to present the images for a fixed duration, to then remove the face image and present the response scale in the centre of the display. With this procedure, we ensured that participants focused on the face stimulus for the same amount of time for each stimulus, allowing for better comparisons between trials. Test runs by first two authors suggested that these durations should vary across stimulus conditions for a comfortable, but not too slow pace of the experiments. Consequently, a 1,500 ms presentation duration was chosen for the small images, 2,500 ms for the larger images, and 4,000 ms for the moving window condition. Based on the work by Willis & Todorov (2006), ratings are not expected to be influenced by these differences. To examine whether eye movements were affected, dwell times were computed for the entire intervals, and for just the first 1,500 ms (the shortest presentation duration), and the time-course of eye movement patterns analysed.

Images were presented on a light grey background. Before the presentation of the images, the standard drift correction target of the Eyelink 1000 system was presented outside the images (for locations, see Fig. 1B). These fixation targets before stimulus onset served to avoid directing attention to a particular area inside the image (Bindemann et al., 2010; Hermens & Walker, 2015) and balancing eye movements starting from the left and the right (Arizpe et al., 2012; Luke & Pollux, 2016). To elicit participants’ responses, a response screen, created in Microsoft Powerpoint, was presented after the face stimulus. This response screen (Fig. 1A) contained the question ‘Trustworthy?’ or ‘Dominant?’ (depending on the instruction), and five response boxes (numbered 1–5), with the labels ‘not at all’ and ‘very much so’ shown next to the one and five box, respectively. A small red square served as the mouse cursor.

### Design

The two judgments (trustworthiness and dominance) were performed in separate blocks, whose order was counter-balanced across participants. Within each block, the 39 images were presented in a random order. There were no practice trials, but due to randomization, we expect any practice effects to cancel out in the average data.

### Procedure

Before taking part, participants provided written consent and received task instructions. Participants were explained that images of faces would be shown, and that the task was to judge these faces for how trustworthy and dominant they looked on the basis of their appearance alone. In the moving window version, participants were informed that they would see a degraded version of the image, but were asked to perform the task as normally as possible. The eye tracker was then set up and calibrated using the nine-point calibration of the eye tracker, which was repeated until the measured eye gaze locations were located on the three by three grid on which the calibration targets were presented (indicating good calibration, associated with a reported 0.25°–0.5° accuracy and a 0.01° RMS resolution). Participants performed two blocks of each 39 trials (one for the trustworthiness task and one for the dominance task). At the end of the experiment, participants were debriefed about the purpose of the experiment and, if they did not have any further questions, thanked and dismissed.

### Data analysis

Mouse responses were analysed on the basis of the horizontal and vertical coordinates of the mouse position when the left mouse button was clicked. A small margin around each of the boxes allowed for mouse-clicks just outside the box. Sporadically, participants clicked further away from a response box. These trials were coded as missing data, and analysed as such. For statistical comparisons, we used mixed effects models (Baayen, Davidson & Bates, 2008; Winter, 2013) instead of more traditional ANOVAs on participants’ averages across trials, incorporating not only variability across participants, but also across items (the individual images), and allowing for sporadically missing data. For these analyses we used the *lme*4 R package (Bates et al., 2015). The reported statistics for these analyses are χ^2^ values (goodness-of-fit measures), which measure the extent to which the fit of an extended model (e.g. one including an interaction term) exceeds that of a reduced model (e.g. one with only main effects).

**Figure 2:**
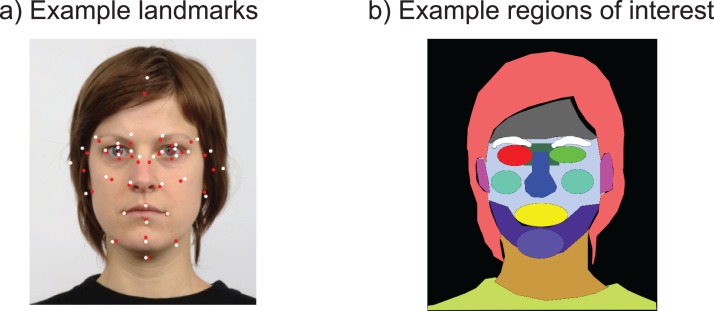
(A) Illustration of the 35 landmarks used across each face, before (red) and after (white) transformation to the average face. (B) Illustration of the regions of interest used.

Ratings were also analysed with respect to facial features, such as the width of the eyes and the thickness of the lips. To obtain these features, 35 landmarks were manually coded for each of the faces (see Fig. 2A) using a custom-built Matlab script asking the user to click on each of these landmarks for each of the face stimuli in the experiment. Custom-built R scripts then derived facial feature measures from these landmarks, which were then used to predict trustworthiness and dominance ratings using multiple regression analyses. Because the main focus of the study was on eye movements, and detailed studies of facial features on a much broader range of stimuli and larger sample of participants have been reported (Vernon et al., 2014), we restricted the number of landmarks coded, as well as the features in these analyses to a selection of measures that could be assumed to be relatively uncorrelated (to avoid issues with multicollinearity, for example, only a measure of the surface area of the eyes was included, but not the width or height of the eyes), and measures that could be directly computed from the facial features with addition, subtraction, multiplication, division operations (i.e. no curve fitting).

Two methods were used to analyse the eye movement data in relation to the task (trustworthiness or dominance ratings). For both methods, the raw eye movement data were first automatically parsed into fixations and saccades using the in-built algorithm of the Eyelink (SR Research, Kanata, ON, Canada) system. In this algorithm, combined 30°/s velocity and 8,000 deg^2^/s acceleration thresholds are used to detect saccades, whereas periods in between the saccades are classified as fixations. In addition, blinks are defined as intervals in which the two measured features of the eye image (pupil centre, corneal reflection) are missing. The remaining intervals are defined as fixations. For the analyses, we only used the fixations.

The first method uses heatmaps aligned to the average face structure to obtain an idea of the regions of interest (ROIs) that may be fixated differently in the two tasks, a method inspired by Cornelissen et al. (2009) (see also, George et al. (2011)) for aligning fixations to human bodies. This method used the landmarks defined earlier, and affine transformations linking the individual landmarks to the average landmark location to transform fixation positions to a common fixation map. The transformed fixations were then combined into heatmaps for each task by superimposing Gaussian kernels around each fixation (kernel width = 1/25th of the screen width, set by trying out a range of values).

For the second method, ROIs were manually coded using the Gimp software by colouring the area of the image deemed to belong to certain regions (e.g. the left eye, the chin) in a particular colour (by first superimposing a black layer for the background and then a transparent layer for the ROIs onto the image, which were later merged into a single ROI image). Fixations were then superimposed onto these ROI images using a custom built Python script, and classified according to the colour and therefore region of the face they fell onto. Regions were defined for the two eyes, the area between the eyes, the nose, the mouth, the cheeks, the eye-brows, the chin, the hair, the ears, the jaw, the forehead, the neck, the clothing, the remainder of the face, and the background (‘Elsewhere,’ see Fig. 2B). Dwell times, the number of fixations and the first area fixated were computed for these regions, as well as fixations on these areas over time (time-course analysis).

## Results

### Ratings

#### Internal consistency

Internal consistency of the ratings was generally high. For trustworthiness the Cronbach’s alpha values were 0.89 (small images), 0.79 (large images), and 0.79 (moving window). For dominance the Cronbach’s alpha values were 0.86 (small images), 0.81 (large images), and 0.90 (moving window).

#### Average ratings

**Figure 3:**
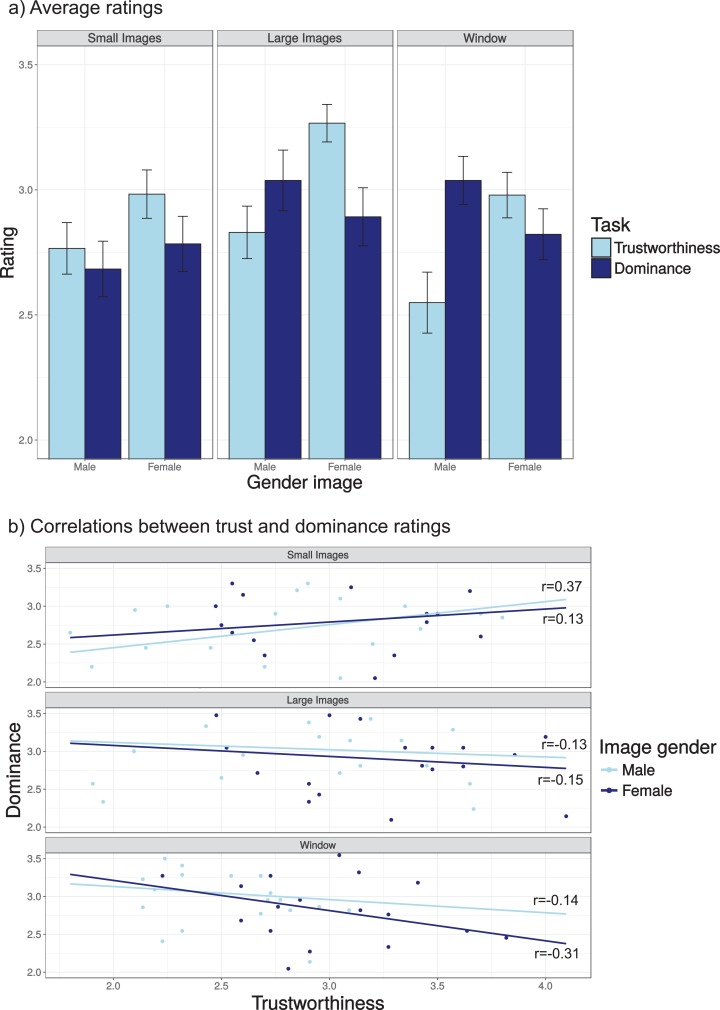
(A) Average trustworthiness and dominance ratings for the three viewing modes; (B) scatter plots examining the association between trustworthiness and dominance ratings. Each dot in these images show the average rating for a face image and lines the best fitting regression lines for that presentation mode and image gender. Numbers in the plots indicate the Pearson correlation for each image gender.

Figure 3A plots the average trustworthiness and dominance ratings for images of male and female models and for the three presentation conditions (small images, large images, moving window). For each presentation condition, females received higher trustworthiness ratings than dominance ratings, whereas for males, the pattern appears to reverse for the larger images. This was reflected in a significant three way interaction between image gender, presentation condition and task (χ^2^(2) = 14.5, *p* < 0.001). For the small images, no interaction between image gender and task was found (χ^2^(1) = 1.20, *p* = 0.27) and no main effect of image gender (χ^2^(1) = 1.46, *p* = 0.23), but there was a main effect of task (higher trustworthiness than dominance ratings, χ^2^(1) = 7.14, *p* = 0.0075). For the larger images, there was a significant interaction between task and image gender (χ^2^(1) = 28.4, *p* < 0.001), and significant effects of task for female (higher trustworthiness than dominance ratings, χ^2^(1) = 22.6, *p* < 0.001) and male (higher dominance than trustworthiness ratings, χ^2^(1) = 7.92, *p* = 0.0049) images. For the moving window condition, there also was a significant interaction between task and image gender (χ^2^(1) = 41.5, *p* < 0.001), and significant effects of task for female (higher trustworthiness than dominance ratings, χ^2^(1) = 4.98, *p* = 0.02) and male (higher dominance than trustworthiness ratings, χ^2^(1) = 48.0, *p* < 0.001) images.

#### Correlations

**Figure 4:**
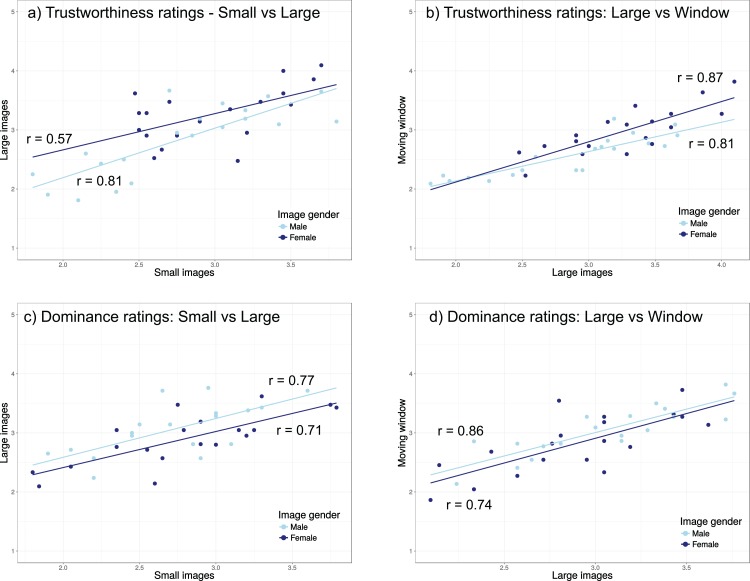
Correlations between the trustworthiness (A, B) and dominance (C, D) ratings for the different viewing modes and image genders. The numbers inside the plots indicate the Pearson correlation for each of the comparisons. All correlations, except for the correlation of 0.57 were statistically significant after correction for multiple comparisons.

To examine whether our findings match past results suggesting that trustworthiness and dominance are largely independent (Oosterhof & Todorov, 2008; Todorov et al., 2008), we examined the association between these two rating types for each of the images (Fig. 3B), showing no significant associations (Pearson correlations, uncorrected *p*-values all above *p* = 0.11). The averages in Fig. 3A suggest that the ratings in the three presentation modes are largely similar (except the male trustworthiness ratings for small images), but to examine this in more detail, we also examined the association between ratings under the three viewing conditions using the averages per image (Fig. 4). Correlations between ratings in the different conditions were all significant after correction for multiple comparisons, except for the comparison between female faces for small and large images (uncorrected *p*-value of 0.011).

#### Prediction

**Figure 5:**
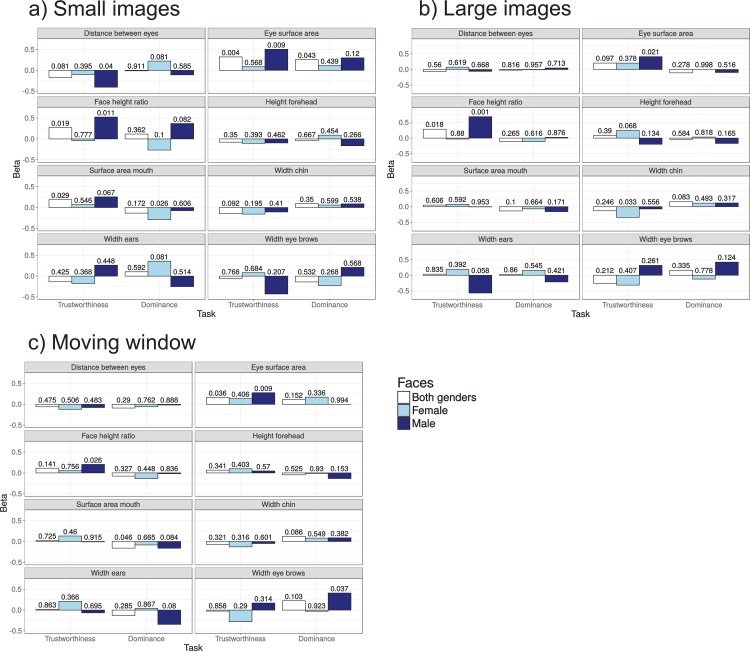
(A–C) Standardized regression weights and associated *p*-values from a multiple regression model predicting trustworthiness and dominance ratings on the basis of facial features.

To examine which facial features can predict participants’ trustworthiness and dominance ratings, Fig. 5 and Table 1 provide an overview of the results of multiple regression analyses linking facial features to trustworthiness and dominance ratings. To examine whether predictions were different for male and female faces, estimates were obtained for three different models: One based on all images (including face gender as a predictor variable, beta coefficient not shown), one for male images only, and one for female images only. The coefficients Fig. 5 represent the weights for the different facial features in the regression model after standardizing and centring of the variables (allowing for direct comparisons of the coefficients). The overall model fits, listed in Table 1 are based on a 10-fold cross-validation procedure (using the R *caret* package). The *R*^2^ values of around 0.50 suggests that around 50% of the variance in the ratings can be explained from the facial features, with slightly better predictions for trustworthiness ratings for male faces. Larger eye surface areas and a larger height-to-width face ratios resulted in higher trustworthiness ratings, particularly for males and under normal viewing conditions (i.e. without a moving window). Overall, prediction was not as good as in past work (Vernon et al. (2014), who achieved 58% of the variance explained), which could be due to the use of standardized images in the present study, which may have restricted the range of facial features (e.g. the surface area of the mouth, which is higher for smiling faces). This would be in line with the lack of a significant prediction for mouth surface area in the present study, whereas mouth features were strong predictors of trustworthiness in the past study (Vernon et al., 2014). Alternatively, the lower performance could be due to fewer facial features used in the present predictions. Interestingly, whereas eye surface area was positively correlated with trustworthiness in the present study, it was negatively correlated with trustworthiness ratings in the past study (Vernon et al., 2014).

**Table 1:** Overall regression model fits when predicting trustworthiness and dominance ratings using a weighted combination of facial features (shown in Fig. 5).

Small images				
Task	Gender image	RMSE	MAE	*R*^2^
Trustworthiness	Male	0.51	0.45	0.68
Trustworthiness	Female	0.58	0.53	0.48
Trustworthiness	Both genders	0.54	0.45	0.46
Dominance	Male	0.72	0.66	0.42
Dominance	Female	0.53	0.47	0.71
Dominance	Both genders	0.53	0.47	0.27

Large images				
Task	Gender image	RMSE	MAE	*R*^2^
Trustworthiness	Male	0.54	0.46	0.73
Trustworthiness	Female	0.53	0.48	0.50
Trustworthiness	Both genders	0.59	0.52	0.41
Dominance	Male	0.51	0.46	0.57
Dominance	Female	0.70	0.64	0.26
Dominance	Both genders	0.48	0.43	0.36

Moving window				
Task	Gender image	RMSE	MAE	*R*^2^
Trustworthiness	Male	0.34	0.30	0.63
Trustworthiness	Female	0.52	0.47	0.24
Trustworthiness	Both genders	0.41	0.33	0.42
Dominance	Male	0.40	0.35	0.70
Dominance	Female	0.60	0.54	0.48
Dominance	Both genders	0.42	0.35	0.51

### Eye movements

#### Heatmaps

**Figure 6:**
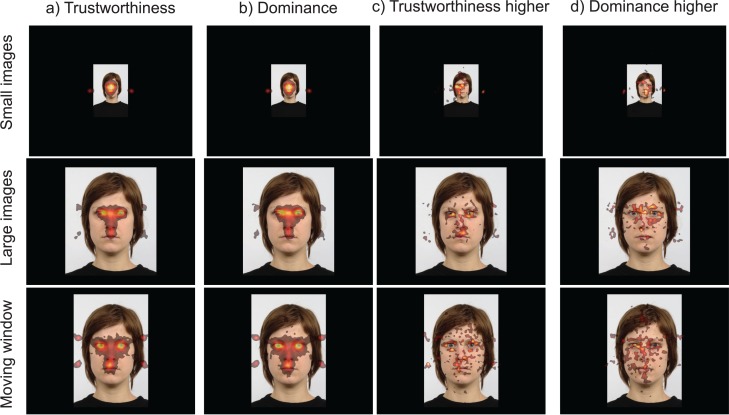
(A and B) Heatmaps of fixations across the three viewing modes (small images, larger images, moving window) and tasks (trustworthiness and dominance ratings); (C and D) difference maps showing where more fixations were made for trustworthiness ratings than for dominance ratings, and vice versa.

To examine whether patterns of eye movements differed across tasks and presentation modes, heatmaps (Fig. 6) were created from fixations aligned to an average face (for each presentation mode) based on the 35 landmarks coded for each face stimulus (Fig. 2A). To create these heatmaps Gaussian kernels around each fixation with a width of 1/25th of the screen width were pooled into a map (using an adaptation of the Pygaze software, Dalmaijer, Mathôt & Van Der Stigchel (2014)). Heatmaps provide an intuitive way of inspecting fixation patterns, independent of pre-defined ROIs. On the downside, there are many choices to be made when creating heatmaps (e.g. kernel width, whether to use fixation counts or fixation durations), which makes comparison across studies complicated (Bojko, 2009). Statistical comparison of heatmaps is possible, but complicated and debated (Caldara & Miellet, 2011; Lao et al., 2017; McManus, 2013; Miellet, Lao & Caldara, 2014), and for these reasons, we limit our current discussion to a graphical interpretation.

In line with past work (Barton et al., 2006; Walker-Smith, Gale & Findlay, 1977), we find that fixations are centred around the eyes, nose and mouth. A broader distribution of fixations was found for the smaller images (in line with Guo (2013)), and for the moving window condition. The broader distribution for the smaller images could be due to the size of the heatmap kernel compared to the face image. The broader distribution of fixations for the moving window, in contrast, cannot be explained from differences in the relative kernel size (about the same as for the larger images condition), or the presentation duration (because it was maintained when only the first 1,500 or 2,500 ms of the trial was used, see images on Hermens (2017)). This suggests that the lack of extrafoveal vision led to a broader exploration of the image, although the inspected areas are still very close to the eyes, nose, and mouth (hair, cheeks, and chin were not often fixated). To examine whether the distribution of fixation depends on the task, difference heatmaps were created (indicating where more fixations were placed for trustworthiness than for dominance, or more for dominance than for trustworthiness, Fig. 6, right columns). These difference maps do not provide a clear pattern of results for the smaller images and the moving window condition. For the larger images, more fixations appear to be aimed towards the area below the eyes for trustworthiness ratings, and the area above the eyes (including the eye-brows) for dominance ratings.

#### First fixated area

**Figure 7:**
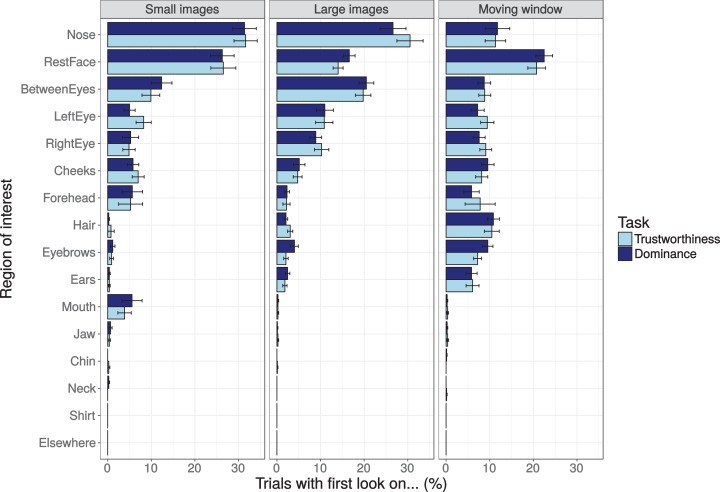
Percentage of trials with a first look on the different regions of interest (ROIs) inside the face image after image onset for the three presentation modes (small images, larger images, and moving window) and the two tasks (trustworthiness ratings, dominance ratings). The ROIs are ordered by the mean percentage across the viewing conditions and tasks (most often fixated areas presented first). Error bars show the standard error of the mean across participants.

Using the ROIs, the area first fixated after the fixation point (landing ROI of the first saccade into the face region) was compared across the three presentation modes and two tasks (Fig. 7). These first look percentages should be relatively independent of the presentation duration of the stimuli (which varied across presentation modes), and may be important for first impressions, as past studies have suggested that such impressions are made in the first 100 ms of stimulus presentation (Willis & Todorov, 2006) and that for face recognition two fixations suffice (Hsiao & Cottrell, 2008). The percentages of first looks suggest that participants most often saccade towards the centre of the image (the nose, the rest of the face, and between the eyes). It also suggests that while presentation mode influences the pattern of eye movements (which could relate to variations of the relative location of the fixation points), there is little or no effect of task (trustworthiness or dominance). A mixed effects Poisson regression on the counts failed to converge for the three-way interaction. Mixed effects Poisson regressions for each of the ROIs showed no interactions between task and viewing mode (all uncorrected *p*-values > 0.11), a main effect of task only for the eye-brows (*p* = 0.0086), and significant main effects of viewing mode for almost all ROIs (except for the jaw and chin regions, which already received very few first looks).

#### Dwell times

**Figure 8:**
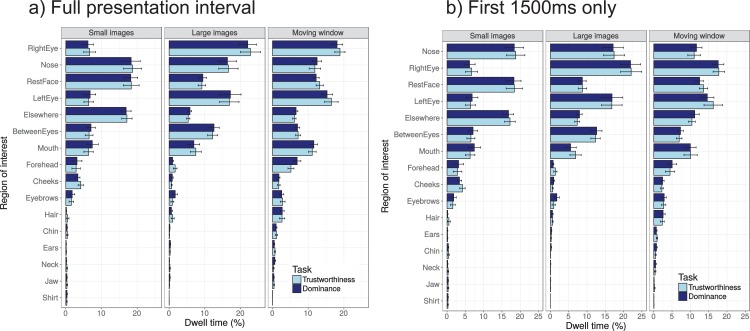
Dwell times on the different regions of interest (ROIs) as a percentage of the total viewing interval, shown for the three presentation modes (small images, larger images, and moving window) and the two tasks (trustworthiness ratings, dominance ratings). The ROIs are ordered by the mean percentage across the viewing conditions and tasks (longest fixated areas presented first). (A) Dwell times across the entire presentation intervals (1,500, 2,500, and 4,000 ms). (B) Dwell times across the first 1,500 ms only. Error bars show the standard error of the mean across participants.

In a second ROI analysis, dwell times on the various regions were examined (Fig. 8). Dwell times measure the total amount of time spent fixating on a particular ROI, and are thought to depend not only on attention allocation, but also the size of the ROI (larger regions tend to attract longer dwell times) and the distance towards the centre of the screen or image (more fixations on the central region of images, due to the central bias, Tatler et al. (2010)). These latter two factors, however, should have little influence on the comparison between the two tasks (the visual input is the same) and the presentation modes (face images the same, although some influence may be possible due to differential cropping of the images). Dwell times tend to be presented as a percentage of the total fixation duration (normalizing for trial duration) for easier comparison across experiments and studies.

We here show dwell times across the entire trial interval (1,500 ms for small images, 2,500 ms for large images, 4,000 ms for the moving window condition, results in Fig. 8A) and across the first 1,500 ms of each trial (Fig. 8B). Across the entire viewing interval, the right eye was fixated for longest, before the nose, the rest of the face, and the left eye, whereas for the 1,500 ms intervals, the nose was fixated for longest (with a very small margin). Dwell times do not seem to strongly depend on whether the entire interval was examined or only the first 1,500 ms, with slightly longer dwell times on ‘elsewhere’ regions when only the first 1,500 ms were used (possibly because of the relatively stronger contribution of the first fixation, which was on the fixation point). We here report the statistics for the first 1,500 ms. For this interval, no interactions were found between task and presentation mode for any of the ROIs (all uncorrected *p*-values > 0.36), and no main effects of task (all uncorrected *p*-values > 0.24), but significant main effects of presentation mode for almost all ROIs, except for the mouth (uncorrected *p*-value = 0.013), the jaw (uncorrected *p*-value = 0.036), and the eyebrows (uncorrected *p*-value = 0.023, all other *p*-values < 0.002).

#### Number of fixations

**Figure 9:**
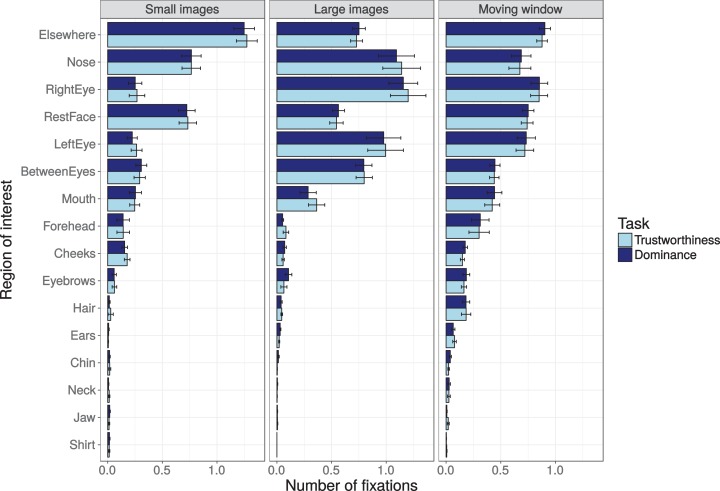
Number of fixations on the different regions of interest (ROIs) within the first 1,500 ms, shown for the three presentation modes (small images, larger images, and moving window) and the two tasks (trustworthiness ratings, dominance ratings). Error bars show the standard error of the mean across participants.

Dwell times are combination of the number of fixations on an area and the duration of each of these fixations. While dwell times and number of fixations are often related, they can show subtle differences. Figure 9 shows the number of fixations on each of the ROIs per task and presentation condition (across the first 1,500 ms of the trial). Interestingly, the ‘elsewhere’ ROI was fixated most often, while dwell times on this area were relatively short (Fig. 8), meaning that individual fixation durations on this area are short on average. Poisson mixed effects regression analyses on the counts showed no ROIs with an interaction between task and experiment that survived Bonferroni correction for the number of comparisons (smallest uncorrected *p*-value = 0.023, for the ‘mouth’ ROI), and no ROIs with a significant main effect of task (smallest uncorrected *p*-value of 0.023, for the ‘chin’ ROI). The effect of presentation mode, in contrast, was significant for all ROIs (largest *p*-value = 0.0060, for the ‘jaw’ ROI). This means that while the relative importance of the ROIs differs between the number of fixations and the dwell times, the effects of task and presentation mode are essentially the same.

#### Time-course

**Figure 10:**
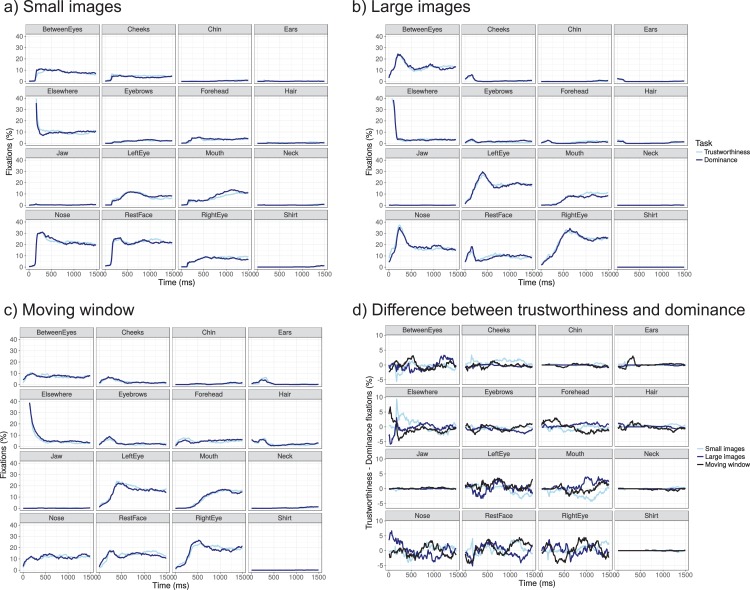
(A–C) Time-course of fixations towards the different regions of interest for the three presentation modes (comparing trustworthiness and dominance ratings), and (D) the difference between trustworthiness and dominance fixations across three presentation modes.

Past work varying the presentation time has suggested that 100 ms suffices to form a first impression (Willis & Todorov, 2006) and that two fixations suffice for face recognition (Hsiao & Cottrell, 2008). Dwell times pool possible differences between tasks across the entire presentation interval, and may therefore obscure task effects that arise, for example, early in the trial (but may be compensated for later on in the trial). To examine whether task effects may be present during certain parts of the trial period, Fig. 10 plots the time course of fixations on each of the ROIs for the two tasks, and the difference between the two tasks (Fig. 10D) for each presentation mode. For all three presentation modes, curves start at the ‘elsewhere’ region (the fixation point). They then move to the nose, between the eyes, elsewhere on the face, before moving to the eyes, and later on, to the mouth area. No clear or systematic differences are found between the two tasks (trustworthiness or dominance), in line with the first area fixated, dwell times and the number of fixations.

#### Individual differences

**Figure 11:**
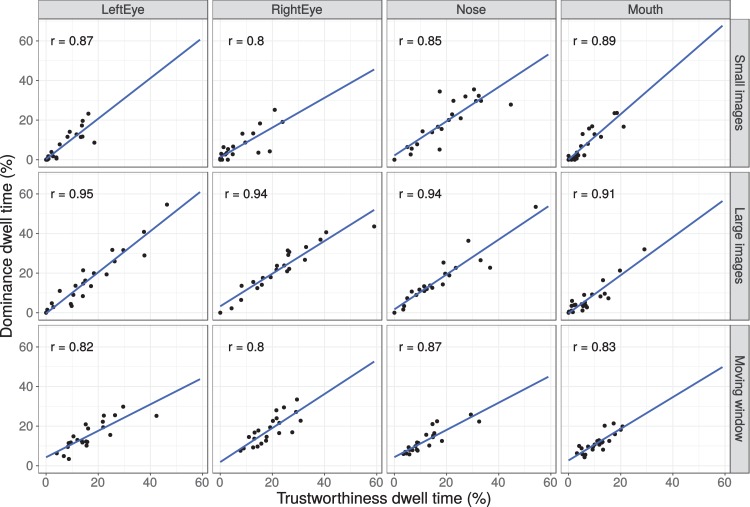
Dwell times per participant for trustworthiness and dominance ratings on the four main areas (left eye, right eye, nose, mouth) and the three presentation modes. Numbers inside the graph show the Pearson correlation.

Studies have suggested that eye movement patterns to face stimuli vary substantially between participants, but are consistent within participants (Mehoudar et al., 2014; Peterson & Eckstein, 2013). In order to examine whether such individual, but consistent differences are also found in the present setting, Fig. 11 plots the dwell times to four main ROIs for the two tasks (trustworthiness and dominance). While dwell times on the different ROIs vary substantially across participants, dwell times within participants are highly consistent across the two tasks, with correlations varying between 0.80 and 0.95 (all *p*-values < 0.001).

#### Classification

In a final analysis, we examine how well participants’ ratings or the task that participants were performing can be predicted from the pattern of eye movements. A broad range of analyses can be performed in this context, varying the eye movement parameters used (e.g. dwell times, first region fixated, number of fixations, or sequences of fixations), and the ROIs included. The present analysis served to get a broad idea of how well ratings and tasks can be predicted from eye movement patterns (previously, in the “prediction” section we already examined how well ratings could be predicted from facial features, independent of how long they were fixated).

**Figure 12:**
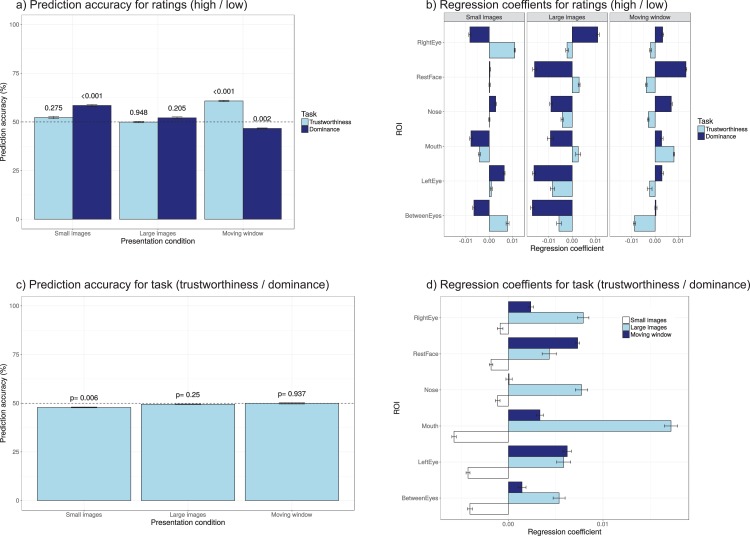
(A and B) Prediction accuracy and regression coefficients for prediction of ratings (low or high) on the basis of dwell times; (C and D) prediction accuracy and regression coefficients for the prediction of task on the basis of dwell times. Error bars show the standard error of repeated runs of the prediction with different non-overlapping random samples of participants for the training and test set. The numbers above the bar indicate the results of one-sample *t*-tests to examine whether the accuracy differed significantly from chance (50%), uncorrected for the number of comparisons.

For the analysis we used dwell times on the six ROIs with the longest dwell times (left eye, right eye, mouth, nose, the rest of the face, and between the eyes) to avoid problems with model estimation due to near zero values. In order to use a mixed effects logistic regression (which provides details on performance accuracy over variance explained), the ratings were split into low (one and two) and high (four and five) ratings. The mixed effects logistic regression was first trained on a random subset of 15 participants, and then tested on the remaining participants in the sample. The resulting accuracy was computed from 10 repeated runs of this procedure (the computations were fairly slow, and results highly consistent across runs). Figure 12 shows the results. For some of the conditions, the prediction of the ratings (high or low) was above chance (dominance for small images, trustworthiness for the moving window condition). Task could not be predicted above chance from the dwell times, in line with the absence of task differences in the dwell times plots (Fig. 8).

The coefficients indicate whether longer dwell times on an area (positive coefficients) led to more frequent high ratings, or were indicative of the trustworthiness task (bottom of Fig. 12). For the prediction of ratings, the sign of the coefficients (direction of the effects) was not consistent across presentation modes or tasks. For the prediction of task, the coefficients were consistent within presentation mode (suggesting that participants more often looked elsewhere for trustworthiness ratings and for small images, and more often to the main ROIs for dominance ratings for the larger images), but did not lead to accurate performance.

## Discussion

Studies have suggested that first impressions of other people’s faces are based on two main dimensions: Trustworthiness and dominance. Analysis of features of faces with high and low ratings have suggested that the shape of the mouth may be important for trustworthiness ratings, whereas features that indicate masculinity (e.g. eyebrows, chin, cheeks) are important for dominance ratings (Oosterhof & Todorov, 2008; Vernon et al., 2014). In the present study, we examined whether people, when rating faces, visually inspect these specific regions. To determine the robustness of these ratings across how faces are perceived (e.g. seen from a distance, nearby, or with a restricted extrafoveal view), different viewing modes were used with small faces simulating a large viewing distance, large faces simulating face-to-face viewing, and a moving window examining the role of extrafoveal visual information. We focused on trustworthiness and dominance ratings, because (1) these two dimensions have been shown to be largely independent constructs, (2) past studies have identified facial features that are important for these tasks. The results suggest that the viewing patterns (and the ratings, but to a smaller extent) depend on how the faces are presented, but no clear effects of task (trustworthiness or dominance ratings) were found. Participants showed large individual differences in how long they inspected each of the areas of the face, but these differences were highly consistent across the two tasks.

Participants’ eye movements tended to focus on the eyes, nose, and mouth regions of the face, as well as the region between the eyes and elsewhere on the face. Analysis of the first fixation on the face, the dwell times on the ROIs, and number fixations on the ROIs, as well as the time-course, did not find systematic effects of the task performed (trustworthiness or dominance ratings), and no adequate predictions of the task could be made from the dwell times. Eye movement patterns, however, were highly variable across participants, but very consistent between tasks. This means that each participant uses their own method of inspecting the stimuli, but does not vary this method across tasks. Our results are therefore in line with past studies that did not find an effect of task on eye movement patterns towards faces (Kwart, Foulsham & Kingstone, 2012; Nguyen, Isaacowitz & Rubin, 2009; Pelphrey et al., 2002) and studies that showed large individual differences in eye movements towards face stimuli (Mehoudar et al., 2014; Peterson & Eckstein, 2012). A possible reason for the absence of a task effect could have been a lack of statistical power to detect significant differences. However, because task did not interact with the presentation mode, the effect of task could be considered across all three experiments. When all three presentation modes are considered, the sample size sufficed according to recent simulations with the type of analysis that we used (mixed effects, Brysbaert & Stevens (2018)). Moreover, we did find highly significant effects of stimulus size and the use of the moving window, which suggests that some differences in eye movement patterns towards faces can be detected with the sample sizes used. Most studies that found task effects on eye movements towards faces used an identity task as one of the tasks (Armann & Bülthoff, 2009; Malcolm et al., 2008; Schurgin et al., 2014), with one exception (where trustworthiness and happiness were compared, Calvo, Krumhuber & Fernández-Martn (2018)). Two of the studies that did not find differences used attractiveness (Kwart, Foulsham & Kingstone, 2012; Nguyen, Isaacowitz & Rubin, 2009), whereas Pelphrey et al. (2002) used free viewing and emotion judgments. While this would need to be explored further, it may be that eye movements for identification differ from those for first impressions.

Past studies have suggested that task can be predicted from eye movements if certain methods are used (e.g. hidden Markov models, Borji & Itti (2014)), but not for all methods (Greene, Liu & Wolfe, 2012). It may be the case that in our analysis, we did not sufficiently explore all available eye movement measures, and machine learning and classification methods (we have made our data available in Hermens (2017), allowing for further exploration). In the application of the methods in the present context, it was important to take into account the repeated measures across participants and images, which limited the range of methods (and thereby the levels of the dependent variable) that we could use. We could have averaged the data across stimuli or across participants (like we did to examine the prediction of ratings from facial features). While averaging made sense for facial features (since each face only has one value for each feature), averaging both dwell times and ratings would have removed important information from the dataset. Considering the original research question, namely whether participants visually inspect important regions for each of the two tasks (e.g. the mouth area for trustworthiness ratings), the poor classification results from dwell times suggest that observers do not systematically inspect these regions. Instead, what is more likely is that, because we only used face stimuli with a neutral expression, participants were not particularly drawn to certain areas of the faces (the mouth, for example, mostly conveys trustworthiness when it is smiling; Calvo, Krumhuber & Fernández-Martn (2018)).

Although our study focused specifically on eye movement patterns, we could also analyse ratings to examine whether we were able to replicate earlier findings in this domain (Oosterhof & Todorov, 2008; Vernon et al., 2014). We found that ratings were consistent (high Cronbach alpha’s and correlations across the viewing modes), but did not correlate strongly between the two tasks (trustworthiness and dominance). The consistent ratings across viewing conditions is particularly interesting given how different the view of the image was in the moving window presentation. This could relate to the finding that for face identity, two fixations suffice (Hsiao & Cottrell, 2008), although by restricting the number of fixations, participants could adopt strategies of finding the optimal fixation position and maintaining fixation for longer. Possibly, the similar judgments when extrafoveal vision was restricted could be due to the relatively large window used (around 4.5° of visual angle), which is beyond the fovea (Rayner, 1998), or the use of the degraded image for guidance as to where to find the image, which may have provided clues for the judgments. The relative stability of the ratings across presentations is in line with Bryan, Perona & Adolphs (2012), who showed that (for trustworthiness ratings) it is the distance at which the photograph is taken that matters, but not the size of the image on the screen.

Average ratings differed between male and female faces, with higher trustworthiness ratings than dominance ratings for female faces, but higher dominance ratings than trustworthiness ratings for males (but not for small images). These gender differences also persisted into the regression analysis that tried to predict ratings from facial features. For male faces, the face height ratio and the surface area of the eyes predicted trustworthiness ratings, but no such effects were found for female faces. Overall, around 50% of the variance in the ratings could be predicted from the facial features, which was lower than found in past work (58%, Vernon et al. (2014)). This past study, however, had a much larger set of face stimuli (*N* = 1,000), a larger range of ratings (1–7), and a larger number (*N* = 65) of facial features (Sutherland et al., 2013; Vernon et al., 2014). The present study used fewer stimuli to avoid long testing sessions in the eye tracker. More importantly, the past study employed a broad range of stimulus pictures (‘ambient images’), which are likely to have more distinctive features than the uniform face database images (Langner et al., 2010) used for the present study. Particularly important may have been the lack of emotions in the face stimuli, because past work has suggested that smiling faces yield higher trustworthiness ratings than angry looking faces (Nurmoja & Bachmann, 2014; Oosterhof & Todorov, 2008; Sutherland, Young & Rhodes, 2016; Willis, Palermo & Burke, 2011). The neutral expressions in our stimuli may have led participants to look for other cues than a smiling expression, which could have led to the focus on the eyes rather than the mouth. Future work should therefore examine to which extent the results depend on the range of facial expressions.

In the heatmaps, we found relatively few systematic task effects. For the larger face images, the results were clearest, and suggested that participants fixated just below the eyes for the trustworthiness judgments and slightly above the eyes for dominance ratings. The modest task effects on the heatmaps was unexpected as the heatmap method had been suggested to be more powerful than a ROIs approach (Cornelissen et al., 2009; George et al., 2011), because it does not rely on the particular choice of the ROIs (Caldara & Miellet, 2011). A possible reason may be the remapping of fixations across images. Earlier work used computer-generated images (Cornelissen et al., 2009; George et al., 2011), which may be easier to map onto a single image than photographs. A further reason may relate to the visualization method. Earlier work (Cornelissen et al., 2009; George et al., 2011) used a grid approach, counting the number of fixations falling in each section of the grid. The present work applied a heatmap approach, where a 2D Gaussian was fitted around each fixation, although it is not immediately clear how this difference may affect the results.

The heatmap analysis also showed that the focus of attention was very strongly centred around the eyes, nose, and mouth (Barton et al., 2006; Walker-Smith, Gale & Findlay, 1977), and less so on areas thought to be important for the dominance ratings (e.g. eyebrows, jaw, cheek bones). As a consequence, any task differences in dwell times for these areas related to dominance depend on a small number of fixations, making it more difficult to detect differences. For the larger images, participants appear to focus slightly more often below the eyes for trustworthiness ratings and slightly above the eyes for dominance ratings (but not as high as the eyebrows), but it is unclear why this may be the case. This subtle difference, if it would be replicated in future studies, shows the power of the heatmap approach, because standard ROIs would not normally code for areas just below or above the eyes.

The present investigation has some limitations that could be explored in future studies. First, we used a relatively small set of standardized face images. In particular, the use of faces with neutral expressions only could have led participants to focus on other features than the mouth than when a broader range of facial expressions would have been used (Sutherland et al., 2013; Vernon et al., 2014). Second, we restricted our analysis of the facial features to those representing the structure of the face, not the texture, luminance or colour. Again, this may be of more interest if a broader range of images is used, photographed under different conditions (Sutherland et al., 2013; Vernon et al., 2014), but it would be an important area to investigate, as past studies have suggested that texture features may play a role for dominance ratings (Sutherland et al., 2013; Tsankova & Kappas, 2016) as well as for trustworthiness ratings (with brown eyes yielding higher trustworthiness ratings, Kleisner et al. (2013)). Third, the matching of the stimuli across the three stimulus conditions (small images, large images, moving window) was not as precise as it could have been. In the smaller images, the clothing of the models was partly visible, whereas in the larger images, this part was removed to increase the size of the face section on the screen. Because participants did not often fixate the clothing of the models, however, we do not expect the presence or absence of this section of the images to have strongly influenced the findings, but future studies aiming to study eye movement patterns towards face stimuli of different sizes should consider keeping the image constant and only vary the resolution on the screen. Future research should also consider using a broader age range in the observers. Studies have suggested that younger and older participants differ in their judgments of trust (Castle et al., 2012; Éthier-Majcher, Joubert & Gosselin, 2013). Other studies have also used a more homogeneous group in terms of gender (Sutherland et al., 2013; Vernon et al., 2014). Past work has suggested that females are more strongly influenced by facial features of trustworthiness (Wincenciak et al., 2013). It would therefore be interesting to investigate whether such demographic differences are linked to differences in eye movements for the two tasks. For other group comparisons and judgments, it has been suggested that a difference in eye movement pattern underlies the difference in judgments. For example, emotion recognition in autism (Pelphrey et al., 2002) and social phobia (Horley et al., 2003) may be related to differences in eye movements.

## Conclusion

The present study shows that while participants are consistent in their ratings of faces for trustworthiness and dominance, have highly individual patterns of eye movements, they do not vary their eye movements across tasks. The results do suggest that how faces are presented (small images, large images, with or without extrafoveal vision) has a strong effect on eye movements of patterns, which is important to take into account in future studies of eye movement patterns towards facial stimuli.

## Additional Information and Declarations

### Data Availability

The following information was supplied regarding data availability: Hermens, Frouke. 2018. ‘Eye Tracking Trustworthiness.’ Open Science Framework. August 13. https://osf.io/3wabp/.
